# MicroRNA Networks in Cognition and Dementia

**DOI:** 10.3390/cells11121882

**Published:** 2022-06-09

**Authors:** Grace S. Blount, Layton Coursey, Jannet Kocerha

**Affiliations:** Department of Chemistry, Georgia Southern University, Statesboro, GA 30460, USA; gb08131@georgiasouthern.edu (G.S.B.); lc15910@georgiasouthern.edu (L.C.)

**Keywords:** miRNA, COVID-19, Alzheimer’s, FTD, vascular, dementia, noncoding, therapeutics, biomarkers

## Abstract

The change from viewing noncoding RNA as “junk” in the genome to seeing it as a critical epigenetic regulator in almost every human condition or disease has forced a paradigm shift in biomedical and clinical research. Small and long noncoding RNA transcripts are now routinely evaluated as putative diagnostic or therapeutic agents. A prominent role for noncoding microRNAs in the central nervous system has uncovered promising new clinical candidates for dementia-related disorders, treatments for which currently remain elusive even as the percentage of diagnosed patients increases significantly. Cognitive decline is a core neurodegenerative process in Alzheimer’s Disease, Frontotemporal Dementia, Lewy body dementia, vascular dementia, Huntington’s Disease, Creutzfeldt–Jakob disease, and a significant portion of Parkinson’s Disease patients. This review will discuss the microRNA-associated networks which influence these pathologies, including inflammatory and viral-mediated pathways (such as the novel *SARS-CoV-2 virus implicated in* COVID-19), and their current status in clinical trials.

## 1. Introduction

There has been an estimated increase of more than 140% in deaths from Alzheimer’s Disease (AD) from the year 2000 to the present [1]. Cognitive decline is a core or co-existing symptom of a range of other diagnoses, including Frontal Temporal Dementia (FTD), Parkinson’s Disease (PD), Huntington’s Disease (HD), vascular dementia, Lewy Body Disease, and Creutzfeldt–Jakob Disease [2]. Genetic, epigenetic, and lifestyle factors can all contribute to cognitive impairment and onset of dementia. The race to find a cure or effective therapeutics has centered around a few key hypotheses with varying success, including formation of brain lesions or plaques from toxic aggregation of beta-amyloid peptides [3]. More recently, the investigation of mRNA vaccines to slow or prevent cognitive decline is an intensifying focus of the scientific and medical communities [4]. 

Effective options for early diagnosis and medical intervention with dementia remain limited. Even as symptoms appear, detection of neuropathology associated with dementia is not definitive with current brain imaging capabilities, although the accuracy and sensitivity of diagnosis improves when correlated with cognitive and neuropsychological tests. A multi-faceted approach to the clinical care of AD and other related pathologies will likely necessitate innovative developments in imaging, accessible biomarkers, therapeutics, and even personalized genetic sequencing. Advances in PET scans to detect Tau [5], the other hallmark protein implicated in AD along with beta-amyloid [3], could significantly enhance detection and monitoring of neuropathological changes in the brain in both pre- and post-symptomatic cases.

The identification of therapeutics for neurodegenerative disease is an ongoing challenge, in part due to the blood–brain barrier (BBB) preventing a majority of small molecule drugs from entering the brain. Those that are excessively large, polar, or highly charged are often impermeable [6]. Many molecules capable of passive transport are excluded by efflux transporters of the ATP-binding cassette (ABC) gene family, which are localized in the BBB [7]. However, the cerebral spinal fluid (CSF) may serve as a viable source of biomarkers, as damage to the brain can impact the levels of proteins and other molecules present in the CSF. For example, low levels of Amyloid beta 42 (Aß42) peptide along with high levels of tau protein in the CSF may indicate AD pathology. Several of the same CSF changes are seen in serum and plasma, which offers additional opportunities as it is less invasive to obtain patient blood samples.

It is now known that noncoding RNAs (ncRNAs) account for more than 90% of our genome and are ubiquitously expressed throughout the body, including biofluids such as CSF, blood, saliva, and urine [8]. This discovery, made by the Human Genome Project, has revolutionized the search for new therapeutics and diagnostic tools in virtually all human health conditions. Notably, the first small RNA drug was FDA approved in 2018 [9], paving the way for a new era of ncRNAs in clinical medicine. Arguably, microRNAs (miRNAs) are the most well characterized class of ncRNAs to date, although the pleiotropic nature of miRNAs necessitates an intensive exploration of their mRNA targets and regulatory mechanisms. In this review, we discuss the clinical potential of miRNAs for cognitive decline and dementia. More specifically, we examine the role of miRNAs in various pathways implicated in dementia pathology, such as inflammatory and viral mediated mechanisms, including the recent COVID-19 virus.

## 2. miRNA Regulation of Neuronal Functions and Cognitive Decline

It is now well established that central nervous (CNS) functions are governed by both small and long neuronal ncRNAs. Mature miRNA transcripts, ~22 nucleotides in length, are arguably the most extensively characterized in the brain, and have established roles in learning and memory, synaptic plasticity, neurogenesis, neuronal differentiation, and neuroprotection [10,11,12,13,14,15,16,17]. Our group reported studies that were among of the first in vivo studies to show that specific brain-localized miRNAs control neurobehavioral phenotypes and identified key miRNA–mRNA networks in neurodegeneration as well [10,11,12,13,14,15,16,17]. Importantly, due to the pleiotropic nature of miRNAs, they can putatively target multiple nodes within a single neuronal signaling pathway [15,17]. The robust influence of miRNAs throughout the CNS underscores their expanding role in neuropathogenesis, including conditions associated with cognitive decline.

It has been estimated that about 15–20% of the population 65 and older have mild cognitive impairment (MCI) and that 65% of those patients with MCI progress into dementia [18]. Several groups have started investigating the role of miRNAs in this neurodegenerative progression. A new study used both a mouse model of aging and memory decline and a cohort of patients with MCI to identify a miRNA signature in circulating miRNAs dysregulated with cognitive impairment [19]. The levels of three miRNAs were altered in both mouse and human blood samples, namely, miR-181a, miR-148a, and miR-146a. Another recent report profiled plasma miRNA expression from amnestic MCI patients (aMCI), which primarily impacts memory, and discovered 46 significantly dysregulated miRNAs [20]. A separate study associated miR-107 plasma levels with aMCI [21].

The expression trajectory of miRNAs in patients who progress from MCI into dementia is under investigation as well. Kenny et al., led a five-year longitudinal study of 31 control subjects and 30 patients with MCI, along with 25 retrospective samples from patients with AD [22]. Plasma was collected yearly, and the levels of extracted miRNAs were quantitated by real-time PCR arrays with the goal of identifying miRNA biomarkers that can detect pre-symptomatic to symptomatic stages of AD. All participants were subjected to neuropsychological tests where visual perception, attention, memory, language, and praxis and executive function were assessed. In addition, the authors analyzed 25 retrospective samples from patients with AD. A strong correlation between miR-206 expression and neuropsychological outcomes was reported, with miR-2016 progressively increasing with cognitive decline. Additionally, miR-206 was significantly elevated in the AD population. Thus, miR-206 in blood is a possible biomarker for patients progressing from MCI to AD-like phenotypes. While let-7b was increased in MCI and AD patients, it did not correlate with trends in progressive cognitive decline (see Table 1 for a list of miRNAs discussed in relation to dementia-associated disease).

## 3. miRNAs in Dementia-Related Diseases

### 3.1. Alzheimer’s Disease (AD)

Alzheimer’s Disease (AD) is the most common cause of dementia and is associated with a progressive loss of neurons and cognitive functions. Identifying miRNAs which influence neuronal viability may yield important insight into AD pathogenesis. The let-7 miRNA family reportedly regulates neural stem cell proliferation and differentiation, and exhibits pro-apoptotic activity in the central nervous system [23,24,25,26,27]. Multiple reports have examined the regulation of let-7 members in neurodegenerative disease such as AD. Independent replicate studies discovered that expression of both let7g-5p and let7d-5p is significantly increased in blood samples from multiple AD cohorts [28]. Additionally, a study by Derkow et al., investigated whether brain-localized neurotoxic let-7 transcripts are released into the cerebrospinal fluid (CSF) and could serve as clinical biomarkers [23]. CSF data from patients with AD were compared with those diagnosed with FTD, major depressive episode (MDE) without clinical or neurobiological signs of AD, and healthy controls. Distinct expression profiles of the let-7 family were found in the CSF of the groups studied, except for elevated levels of let-7b and let-7e in both AD and MDE patients. A separate study by Sorensen et al., analyzed the CSF and blood of patients with AD and other types of dementia. In total, they detected 52 miRNAs in the CSF of almost all patients; two (let-7i-5p and miR-15a-5p) were upregulated and one (miR-29c-3p) was down regulated when comparing AD patients to healthy controls [29]. Collectively, these results suggest that abnormal activity of let-7 miRNAs may mediate cognitive and dementia-associated pathology through critical neuronal signaling pathways.

In addition to the let-7 family, other miRNAs which influence neuroprotection and regeneration are associated with dementia. A study by Fu et al., reported that miR-142-5p promotes neuronal synaptotoxicity both in vivo and in vitro [30]. They found that miR-142-5p enhances neuronal viability and inhibits apoptosis through the G protein-coupled receptor BAI3. Correspondingly, downregulation of miR-142-5p levels in an AD mouse model brain may upregulate expression of the BAI3 transcript. Inhibition of miR-142-5p in cultured neurons restored spatial learning and memory. Significant dysregulation of miR-142-5p expression, as well as that of miR590-5p and miR-194-5p, was discovered in blood samples from a cohort of AD patients compared to healthy controls. Previous reports indicate that miR-342 plays a key role in proliferation and differentiation of neural stem cells as well as in neurotoxicity [31,32]. A recent study suggests that levels of miR-342-5p in the plasma may predict the rate of cognitive decline in AD [33]. These results suggest that miRNAs which control key neuronal functions are dysregulated in dementia patients, and that their expression profiles in biological fluid samples can mirror neuropathological outcomes [29].

Diagnosing AD early with non-invasive techniques is an ongoing effort. Exosomes derived from CSF and serum can serve as an accessible source for miRNA profiling in neurodegenerative disease. A study in an AD mouse model (APP/PS1 mice) found that miR-193b expression is significantly decreased in hippocampal neurons, while total exosomal expression of miR-193b was increased [34]. Furthermore, this same group then capitalized on clinical data that showed high expression of the ATP-binding cassette transporter ABCA1 in the CSF of AD patients in order to specifically immunocapture ABCA1-labeled exosomes. ABCA1-labeled exosomal levels of miR-193b were significantly higher in the CSF of patients with mild cognitive impairment (MCI) or AD compared to control subjects. This same group went on to measure the expression of exosomal miR-193b, as well as miRNA-135a and 384, in serum from patients with MCI, dementia of Alzheimer’s type (DAT), Parkinson’s disease with dementia (PDD), and vascular dementia (VaD) [35]. The expression levels of miRNA-135a and miRNA-384 were upregulated and miRNA -193b was downregulated in the serum of AD patients compared to healthy controls. It was found that miRNA-384 was more successful at differentiating between the types of dementia compared to the other two miRNAs. Another study first identified dysregulated miRNAs in brain tissue from AD patients, then for further analysis focused on those from neurally-derived plasma exosomes. Both miR-132 and miR-212 were downregulated in AD patients [36]. Interestingly, another report showed that the miR-132/212 cluster was downregulated in the frontal cortex of patients with MCI as well as those with AD [37]. Collectively, miR-132 and miR-212 are consistently linked to cognitive decline [36,37], and this information could lead to innovations in clinical care for pre-symptomatic and symptomatic dementia patients.

In addition to the blood, serum, CSF, and exosome studies discussed above, Kenny et al., investigated the tear fluid of patients with MCI or AD as a novel source of disease-specific protein and miRNA-based biomarkers. When the tear fluid was collected, protein content was analyzed using liquid chromatography–mass spectrometry and miRNA content was probed using a genome-wide high throughput PCR-based platform. Elongation initiation factor 4E was seen as a unique protein that was detected only in AD patient samples, and total miRNA abundance was concluded to be higher in tears from the AD patients [38]. Furthermore, higher levels of miRNA-200b-5p levels were observed in tear fluid samples from the AD cohort compared to controls, and could serve as another non-invasive source for diagnostic biomarkers.

Insulin resistance is a critical risk factor in AD and Type 2 Diabetes (T2D), two of the leading global health threats. Due to the extensive overlap in signaling between insulin, neurotransmission, and neuroinflammatory responses, an ongoing area of investigation is identifying shared genetic and/or epigenetic factors between AD and T2D [39], including miRNA-based mechanisms. Specifically, a shared response in AD and T2D to miR-1271 regulation was discovered. Two non-canonical receptor tyrosine kinases (RTKs), *Anaplastic lymphoma kinase* (ALK) and Receptor Like Tyrosine *Kinase* (RYK), were reported to target miR-1271 in analysis of post-mortem AD and T2D tissues. ALK and RYK were both downregulated due to miR-1271 and its ability to elevate PAX4, a transcription factor for Grb2 (a downstream adapter) and NOX4 (a common ROS producing factor), through Catenin signaling [40]. It was observed that ALK and RYK were responsible for the degradation of cytoskeleton integrity in both AD and T2D mice. Grb2 and NOX4 levels were elevated in both AD and T2D studies, and were connected to both the RTKs and cytoskeleton degradation.

### 3.2. Frontotemporal Dementia (FTD)

Neurodegenerative diseases such as Frontotemporal dementia (FTD) are commonly associated with behavioral deficits due to atrophy of the frontal and temporal lobes. FTD is often linked to mutations in Microtubule Associated Protein Tau (MAPT tau), progranulin (GRN) and C9orf721 [41,42]. Groundbreaking genetic studies discovered a repeat expansion within the C9orf721 gene in patients with FTD and ALS as well as in ALS patients with FTD [43,44]. A recent study examined the plasma miRNA signature in a cohort of fifteen FTD, four FTD/ALS, and three ALS patients [45]. Two miRNAs, miR-34a-5p and miR-345-5p, showed increased expression, and two were downregulated, miR-200c-3p and miR-10a-3p. Notably, miR-34a-5p was upregulated in presymptomatic carriers with the C9orf721 mutation, suggesting the mutation influences miRNA levels. Additionally, levels of miR-345-5p progressively increased from healthy controls to presymptomatic C9orf721 in symptomatic patients.

Mutations in the charged multivesicular body protein 2B (CHMP2B) gene are of particular interest due to their association with FTD, Amyotrophic lateral sclerosis (ALS), and early-onset AD. Gascon et al., reported that mice with forebrain-specific expression of the mutant CHMP2B exhibit FTD-related behavioral phenotypes. More specifically, they identified a molecular mechanism involving miR-124 regulation of AMPAR in the mouse model that paralleled results from human frontal cortex and neuronally-derived induced pluripotent stem (iPS) cells of FTD patients [46]. Viral-mediated expression of miR-124 in mice partially restored the imbalance between Ca^2+^ permeable and impermeable AMPARs in the CHMP2B mutant model, as well as associated phenotypic abnormalities. Collectively, this study implicates an AMPAR-miR124 mechanism that could mediate behavioral outcomes in FTD.

Tauopathies are a specific group of neurodegenerative diseases, including FTD, which are associated with abnormal aggregation of the tau protein. Multiple studies have reported a functional role for miRNAs in pathogenesis of tauopathies [47,48]. Kenny et al., used a mouse model (VLW) expressing mutations linked to FTD patients with parkinsonism-17 to evaluate whether pathological expression of tau influences miRNA expression [49]. A significant increase in argonaute-2-bound levels of miRNAs and miRNA expression was detected in the VLW mice, including miRNAs-134, 99a and 101. Notably, one miRNA, miR-29b, was downregulated in the VLW model. Previous reports found that miR-29b expression was decreased in brain tissue from AD patients and that there is a regulatory mechanism between miR-29b and the BACE1 gene [50]. BACE1 is essential for the production of β-amyloid, and its dysregulation is consistently associated with AD pathology [51]. Further studies could support an important clinical role for the miR-29 family in dementia.

Our research group identified a panel of dysregulated miRNAs in FTD patients with specific mutations in the disease-associated progranulin gene [17]. Moreover, distinct miRNA expression patterns were discovered between FTD patients with and without progranulin mutations. Brain region-specific studies offered further insight, as these genetic mutations are present throughout the central nervous system. Collectively, robust miRNA–mRNA regulatory mechanisms may facilitate the identification of sporadic versus genetically-linked neurodegeneration and dementia.

### 3.3. Vascular Dementia (VaD)

Vascular Dementia (VaD) symptoms are symptoms of progressive memory and behavioral deterioration stemming from impaired blood flow, typically followed by AD. rat models used to investigate VaD exhibited advancing cognitive impairment, increased hippocampal neural stem cell senescence (H-NSCs), and decreased neurogenesis. Intravenous injection of embryonic stem cell-derived small extracellular vesicles (ESC-sEVs) significantly rescued the neurodegenerative phenotype. It was discovered that miRNAs endogenously present in the ESC-sEVs restored neurogenesis and reversed senescence through inhibition of the mTORC1 pathway, which includes miR-17-5p, miR-18a-5p, miR-21-5p, miR-29a-3p, and let-7a-5p [52]. These results suggest that ESC-sEVs could be exploited as a potential therapeutic option for VaD. 

A study of plasma samples from a cohort of patients with VaD discovered that miR-29a-3p, one of the miRNAs present in ESC-sEVs, is significantly downregulated compared to healthy controls [53], and that miR-10b* and miR-130b-3p levels were decreased in VaD plasma samples. Notably, the same study discovered a parallel dysregulation of these miRNAs in a cohort of AD samples, with the extent of dysregulation potentially capable of distinguishing AD versus VaD pathology. Examples of a few plasma-based miRNAs that were found to be dysregulated in VaD, AD, and FTD are outlined in Table 2.

### 3.4. Dementia with Lewy Bodies (DLB) 

Dementia with Lewy bodies (DLB) is the second most common form of dementia; it is characterized by memory loss and the impairment of visuospatial, attentional, and frontal executive functions [58,59]. Finding biomarkers for DLB before the onset of symptoms remains elusive; however, miRNAs have the potential to serve as powerful diagnostic or interventional tools. Thanks to the use of machine learning (ML) methods in other studies for disease prevention, ML methods have been applied to the comprehensive miRNA expression of serum samples featuring DLB patients and normal controls (NC), along with gene set enrichment analysis (GSEA). Using a logistic regression method, 180 out of the 216 miRNAs showed a contribution to the set risk prediction model, and seven of those 180 showed importance in the GSEA model [60]. From this, the following miRNAs showed a specifically enriched biological pathway: miR-3122, miR-6861-3p, miR-4298, miR-4298, miR-4728-5p, miR-5698, and miR-1909-5p. The gene targets of these miRNAs were then analyzed and implicated in six signaling pathways: protein kinase signaling, ERK/MAPK signaling, molecular mechanisms of cancer, p38 MAPK signaling, glucocorticoid receptor signaling, and DHA signaling. In particular, docosahexaenoic acid (DHA) is significant because it has been previously reported to be associated with DLB pathology.

Dementia with Lewy Bodies (DLB) is characterized by the deposition of misfolded proteins, the presence of α-synuclein accumulations (Lewy bodies), and in certain cases the accumulation of ß-amyloid senile plaques and hyperphosphorylated tau in the brain [58,61]. DLB shares common characteristics with AD, which can result in misdiagnosis and improper clinical care. A study by Gámez-Valero examined whether miRNA expression profiles from plasma could distinguish between AD, DLB, and healthy controls [62]. Of the 238 miRNAs that were probed, six miRNAs in AD patients were downregulated compared to the DLB patients and healthy controls: hsa-miR-451a, hsa-miR-21-5p, hsa-miR-23a-3p, hsa-miR-126-3p, hsa-let-7i-5p, and has-miR-151a-3p. These miRNAs could enhance sensitivity and/or accuracy of diagnosis and potentially guide treatment plans.

A study by Nelson et al., hypothesized that miRNAs may mediate development of Lewy body pathology in specific brain regions [63]. They examined postmortem tissue from the anterior cingulate (AC) and primary motor (MO) cortex of 52 patients with Dementia with Lewy Bodies (DLB), AD, AD+DLB, and healthy controls. The results of this study showed distinct miRNA expression patterns based on anatomic location (AC or MO). They reported that brain regions with lower susceptibility to Lewy body pathology (MO) have high expression of miR-133b and miR-34a and low expression of miR-137 and miR-7. Collectively, these results suggest that site specific miRNA expression patterns in the brain may predict vulnerability to Lewy body pathology.

### 3.5. Parkinson’s Disease (PD)

One third of patients with Parkinson’s Disease (PD) are diagnosed with dementia as well; therefore, comparing the miRNA profiles of brain tissue from PD patients with and without dementia could identify shared miRNA mechanisms and distinct epigenetic patterns between the two neurodegenerative pathologies. Of the 125 miRNAs that found to be differentially expressed, it was found that a set of 29 miRNAs could be used to classify a PD brain from a healthy brain, 36 miRNAs could be used to differentiate between PD with Dementia and PD without Dementia, and that there a positive relationship exists between miR-10b-5p and age of onset [64].

Another reported study directly compared miRNA expression profiles from blood serum (SER) and cerebrospinal fluid (CSF) of 67 patients with PD, 69 patients with AD, and 78 neurologically normal controls [65]. Extracellular miRNAs detectable in peripheral circulation may provide insight into abnormal molecular changes inside the human brain. The study associated miRNA signals present in cell-free peripheral biofluids with neurodegenerative disease status. Next-generation small RNA sequencing (NGS) was used to analyze miRNA profiles in CSF and SER for the patient cohorts and correlate them with neurodegenerative pathology. Notably, this was the first report to sequence and analyze the miRNA profile of CSF and SER from the same patients. Out of 2228 annotated miRNAs in miRbase (Version 18), 1773 different miRNAs were identified in the CSF and 1757 in the SER. Of these, 41 miRNAs with differential expression in were found in AD CSF; however, far fewer were found when comparing PD patients with healthy controls. The authors noted a few of the miRNAs because of their previous association with neurodegenerative disease, including miRNA-9, miR-34c, miR-34b/c, miR-101, and miR-132. This further supports cumulative evidence that peripheral miRNAs found in the CSF and serum can detect changes in human neuropathology.

### 3.6. Huntington’s Disease (HD)

Huntington’s Disease (HD) is genetic disorder that leads to development of dementia-related symptoms through a progressive decline in thinking and memory [66,67,68]. Our group used the first-ever transgenic primate model for HD to investigate a role for miRNAs in HD pathogenesis and correlated the results with publicly available datasets from human HD brain tissue [15]. By probing miRNA profiles from the frontal cortex of transgenic monkeys expressing mutant huntingtin (HTT) we discovered significant downregulation of miR-128a, which correlated with results from publicly available datasets of human HD brain tissue. Notably, miR-128a was bioinformatically predicted to bind to the HTT and Huntingtin Interaction Protein 1 (HIP1) transcripts, and these miRNA128a-mRNA mechanisms were confirmed in vitro. Additionally, studies in a mouse model showed significant correlation of miR-128 with the CAG repeat mutation that is the genetic cause of HD [69].

A recent study reported significant miRNA editing patterns in HD patients, including A to I and C to U nucleotide changes [70]. Intriguingly, mir-10b-5p was edited to have an additional cytosine at 5’-end, and has previously been linked to HD pathogenesis [71,72]. A target of mir-10b-5p, GTP binding protein 10 (GTPBP10), has been consistently found to be repressed in HD patients. Due to the pleiotropic nature of miRNA transcripts, editing of their sequence could lead to broad alterations in the transcriptome through their target mRNAs.

### 3.7. Creutzfeldt–Jakob Disease (CJD)

Sporadic Creutzfeldt–Jakob disease (sCJD) is a fast, progressive, and untreatable form of dementia that is usually fatal within six months of diagnosis. Pathologically, it creates a porous change in the cerebral grey matter of the brain which causes unusual forms of prion protein (PrP). Rapid diagnosis of sCJD may prove critical if effective treatment becomes available. Norsworthy et al., profiled miRNA expression in blood samples from 57 sCJD patients as well as from 48 healthy control patients [73]. Significantly decreased levels of the miRNAs hsa-let-7i-5p, hsa-miR-16-5p, and hsa-miR-93-5p were detected in sCJD when compared with healthy controls. These results suggest that a peripheral blood molecular profile in sCJD patients may predict diagnosis.

## 4. Mechanisms of miRNAs in Dementia-Related Disease

### 4.1. Inflammatory

Inflamma-miRs are a subset of miRs that modulate the inflammatory process. Because of a systematic pro-inflammatory status associated with AD, these inflamma-miRs have been investigated as a tool to assess the development and progression of AD. The expression of inflamma-miR-17-5p, miR-21-5p, miR-126-3p, and miR-146a-5p was analyzed in plasma samples of AD patients and age- and gender-matched healthy controls (HC) [74]. It was found that miR-17-5p, miR-21-5p, and miR-126-3p plasma levels were significantly higher in AD patients in comparison to HC. In addition, there was an inverse relationship between both miR-21-5p and miR-126-3p and cognitive impairment, and MiR-126-3p was able to distinguish between mild and severe cognitive impairment. The findings of this study suggest that circulating inflamma-miRs can be utilized as tools that are associated with both the development and progression of cognitive impairment in AD.

One report tested the anti-inflammatory medication simvastatin in human AD patients and an AD mouse model [75]. Patients with suspected AD were administered Simvastatin or placebo daily. In addition to cognitive assessments, blood samples were collected to monitor for any significant changes in key biomarkers of the inflammatory signaling cascade, including interleukin-6 (IL-6), interleukin-1 beta (IL-1β), antichymotrypsin (ACT), and human tumor necrosis factor α (TNF-α). Notably, Simvastatin treatment partially restored cognitive deficits in both human patients and in the AD mouse model. The authors found that a regulatory mechanism between miR-10b and a long nCRNA (lnc RNA n336694) may mediate this clinical outcome, suggesting a novel ncRNA–ncRNA signaling pathway in neuroinflammation.

*Bacteroides fragilis* (*B. Fragilis*) is a gram negative bacteria that constitutes a large portion of the human GI tract and produces a unique mixture of neurotoxins, such as the pro-inflammatory lipopolysaccharide BF-LPS. LPS can be seen in the peripheral neuronal nuclei in the brains of patients with AD as well. LPS activates production of the pro-inflammatory transcription factor NF-kB in the human neuronal–glial cells, which induces NF-kB-mediated transcription of pro-inflammatory miRNAs, including miRNA-9, miRNA-34a, miRNA-125b, miRNA-146a, and miRNA-155 [76]. Upregulated expression of these miRNAs influences the transcription of key genes linked to neurodegeneration and plaque formation associated with AD pathology. Overall, these results suggest that neurotoxins released from the GI tract microbiome may play a large role in pro-inflammatory miRNA–mRNA regulatory mechanisms that advance neurodegenerative processes in dementia.

Microglia are natural immune cells found in the central nervous system and are the primary responders in the brain to pathological changes such as the amyloid plaque formations found in AD [77,78]. miRNAs influence microglia function by downregulating the expression of inflammatory effectors when exposed to microglia inflammation. miR-155 is known to have an important role in coordinating immune-related processes through microglia, and is linked to pathogenic outcomes in neurodegenerative disorders such as AD and familial Parkinson’s disease [78,79,80,81]. A study by Aloi et al., invested whether miR-155 expression in microglial cells changes cellular behavior when exposed to fibrillar Aβ1-42 (fAβ1-42) [81]. Notably, it was found that inflammatory signaling impacts the capacity of microglia to catabolize fAβ1-42 through a miR-155 regulatory mechanism, and could thus influence the development of AD pathology.

Reports indicate that miR-22 mediates neuroinflammatory cascades and cell death through pyroptosis [82,83]. Low levels of miR-22 in the peripheral blood of AD patients suggests that mimicking miR-22 function could aid in the reduction of pyroptosis. This hypothesis was tested by injecting mice with a miR-22 mimic, which led to enhanced memory and behavior as well as decreased inflammation [84]. Furthermore, miR-22 could be used to reduce the release of inflammatory cytokines by monitoring inflammatory pyroptosis of glial cells, resulting in increased cognitive function and ability [84]. Of note, studies have shown that miR-22 may function through a signaling axis with several long ncRNAs (lncRNAs), including Lnc-HOTAIR [85] and LncRNA H19 [86].

Bioinformatics studies suggest that risk genes associated with AD are grouped with lipid metabolism and inflammation. The blood–brain barrier is lined with endothelial cells, which have the potential to be mediators of systemic metabolism. This has led to the study of 27-hydroxycholesterol (27-OHC) on microvascular endothelial cell redox state, inflammatory cytokine secretion, and miRNA expression [87]. It was found that the 27-OHC had a regulatory effect on endothelial microvascular cells, which increased the expression of miRNA-933 as well as the secretion of inflammatory cytokines that are known to be elevated in the plasma of dementia patients. Through a miRBase scan, it was further found that miRNA-933 potentially targets brain-derived neurotrophic factor, meaning that miRNA-933 could be evaluated for neuroprotective properties.

### 4.2. Viral

The novel SARS-CoV-2 virus that causes coronavirus disease 2019 (COVID-19) has propelled a global pandemic as a respiratory syndrome over the past few years. Recent efforts investigating the impact of COVID-19 on the central nervous system have gained attention as persisting symptoms of afflicted patients develop. RNA sequencing was performed and discovered twelve differentially-expressed protein-coding genes from the frontal cortex tissue of individuals with dementia compared to patients with COVID-19 and dementia [88]. Using this transcriptome profile, identifying the miRNA targets of differentially expressed mRNAs could uncover novel miRNA–mRNA pathways activated in response to viral infection in the central nervous system. Indeed, a recent study found that the SARS-CoV-2 virus encodes a miRNA, CvmiR-5-5p [89]. Moreover, bioinformatics analysis of the CvmiR-5-5p sequence suggests that it targets genes involved in neurogenesis, neuron development, and neuron differentiation.

The SARS-CoV-2 virus reportedly infects vital organs such as the lungs, heart, kidneys, and brain. It is the third human coronavirus (HCoVs) outbreak in the past twenty years; during 2002–2003, the SARS outbreak occurred, and in 2012 the MERS outbreak occurred. While all three HCoVs are characterized by mild upper respiratory infections, the SARS outbreak showed the potential for high virulence. Along with this, HCoVs have been seen as the cause of neurological disease [90]. This is due to the olfactory bulb, where SARS-CoV can access the brain. Because the SARS-CoV-2 virus is similar to previous generations of HCoVs, namely, SARS and MERS, it has been suggested that SARS-CoV-2 can cause neurological damage. Researchers have theorized that there is retrograde transmission of the virus from the olfactory epithelium to the brainstem, which could impact neurological functions [90]. Angiotensin-converting enzyme 2 (ACE2) serves as a receptor for SARS-CoV-2 and is expressed throughout the CNS. SARS-CoV-2 commonly enters the body through leukocytes, which are then redirected to the bloodstream. In an infected immunosuppressed individual, viremia occurs, which allows the virus to spread from the upper respiratory tract to the CNS; this process takes place due to retrograde axonal transport from peripheral nerves such as the olfactory nerve. Furthermore, it has been demonstrated that a majority of the adverse effects of the virus are due to microglial reactivity caused by neuronal harm as well as inflammatory reactions. In a subsequent study, downregulation of miR-10b, which is regulated by cytokine, was observed in COVID-19 patients, and was correlated with increased levels of the cytokine family members interleukin-2 and interleukin-8 [91]. Based on the collection of these reports, it is possible that miRNAs may influence the neurological response in patients with COVID-19, possibly through pro-inflammatory mechanisms and induction of “cytokine storms”.

A recent study discovered that single-nucleotide polymorphisms (SNP) in miRNA genes can influence patient outcomes in cases of COVID-19 infection. Zhang et al., used genome-wide association studies (GWAS) to discover an SNP in miR-1202 that is associated with COVID-19 and its risk factors, including the presence of dementia-associated pathology, alcohol consumption, HDL, BMI, and even daytime sleeping patterns [92]. Notably, miR-1202 is consistently linked with depression and response to anti-depressants, which have established and significant links to the onset of dementia [93,94].

### 4.3. Gene Networks

Ongoing studies are working to identify translatable miRNA biomarkers that are part of gene networks, including ncRNA–ncRNA interactions. One report used next-generation sequencing from human cerebrospinal fluid (CSF) to find possible therapeutic agents and/or diagnostic markers for AD. It was found that the small ncRNAome (sncRNAome) from exosomes in the CSF was composed of noncoding PIWI-interacting RNAs (piRNAs) and miRNAs, including miR-10a-5p, miR-100-5p, miR-22-3p, mIR-204-5p, and miR-26a [95]. The piRNAs are a small noncoding RNA species that have previously been shown to have the ability to silence repetitive genomic regions, which can equate to genomic stability. This leads to the idea that the piRNAs that were found to be expressed in the CSF in this study regulate gene-expression. The AD patients in this cohort study were previously diagnosed with irregular CSF Aβ 42 and CSF total Tau levels. Due to this prior information, the pTau and Aβ 42/40 ratio was used in a tenfold CV random forest algorithm. The results of this study showed that combining the small RNA signature with pTau and Aβ in a 42/40 ratio aided in analyzing AD dementia patients with high sensitivity and specificity. Furthermore, the same study found that an exosomal ncRNA signature in the CSF can successfully detect AD pathology, including piRNA–miRNA networks.

In an effort to study gene networks of human disease, mice models have been used to provide a cross-species network approach to drug discovery. The mice studied were F1 offspring crossed with TPR50 mice that expressed the human Tau transgene, as tauopathies are an established class of dementia and other neurodegenerative diseases [47,57,96]. The miRNA profiles from the mice were compared to proteomic and transcriptomic data from the human brain to close the species gap. The role of miRNA-203 in neurodegenerative pathways was further validated, as the overexpression of this miRNA repeats miRNA co-expression patterns that are associated with the disease state as well as with induced neuronal cell death. By utilizing a drug-mediated gene expression change database, small molecules with the potential to normalize these disease-associated modules can be identified.

Over the past two decades, advances in genotyping and sequencing have allowed for the identification of genetic variants associated with human complex traits and diseases; however, most of these loci are found in non-coding regions. Because of this, it is challenging to determine mechanisms, as the targets affected by these loci are commonly assigned to either neighboring protein-coding genes or ncRNAs. Using expression quantitative trait locus (eQTL) analysis, these mechanisms can be discovered. The JAMIR-eQTL database uses this method to investigate the correlation between genetic variations and miRNA expression in samples featuring various types of dementia, including AD, DLB, vascular dementia, FTD, normal-pressure hydrocephalus, and mild cognitive impairment [97]. These six types of dementia have been used to identify 2487 cis-miR-eQTLs and 3,155,773 trans-miR-eQTLs, as well as several miRNAs common to multiple types of dementia. Correlating genetic risks with epigenetic mechanisms could identify pathways for sporadic cases of neurodegeneration.

### 4.4. Amyloid-B (Aβ) and Tau

Despite controversy, there is a long-standing hypothesis that early onset Alzheimer’s Disease is a result of the amyloid-β (Aβ) peptide formed from the amyloid beta precursor protein (APP gene). This has fueled the therapeutic goal of reducing the formation of toxic aggregates of Aβ. A subset of miRNAs, such as miR-298, are predicted to have binding sites in the mRNA transcript that encodes Aβ. Recent studies have confirmed that Aβ is regulated by miR-298 through a combination of in vitro and in vivo experiments, including overexpressing miR-298 in an APP mouse model for AD [98]. Furthermore, two single nucleotide polymorphisms (SNPs) were identified in miR-298 that are linked to Aβ levels in AD patients. Collectively, these results support the potential of targeting miRNAs that control toxic formation of Aβ plaques, which are a hallmark of AD neuropathology.

As with Aβ, accumulation of abnormal tau aggregates is a classic pathological feature in dementia-related diseases such as AD. Notably, growing evidence supports independent as well as synergistic mechanisms between Aβ and tau [3,96,99]. Aberrant tau hyperphosphorylation and aggregate formation in AD is reportedly mediated by miRNA networks, as supported by a new report on miR-23b-3p. Jiang et al., showed that miR-23b-3p targets glycogen synthase kinase-3β (GSK-3β) to prevent tau hyperphosphorylation in vitro as well as in an AD mouse model (APP/PS1 mice) [100]. Moreover, in vivo delivery of miR-23-3p restored cognitive decline in the model while inhibiting neuronal apoptosis, Aβ_1-42_ generation, and tau hyperphosphorylation in a neuroprotective role.

Overall, the mechanisms outlined (Table 3) provide a foundation for identifying key miRNA networks in established dementia-associated pathways.

## 5. Clinical Trials

Translation of miRNAs and other ncRNA classes to the clinic is an active and ongoing area of interest; both as diagnostic biomarkers and for medical intervention. The first small RNA therapeutic was approved by the FDA in 2018, unleashing a new era of opportunity for ncRNAs in clinical research. Several active clinical trials are investigating the potential of miRNAs as clinical biomarkers for dementia pathology (Table 4); however, there are no active or closed trials at any trial phase evaluating miRNAs as therapeutics for cognitive decline.

One observational clinical trial focuses on biomarkers of motor neuron disease (MND) and FTD, as they are believed to comprise a neurodegenerative disease spectrum (NCT04961450). The researchers leading this study are collecting samples from a large patient cohort in order to explore diagnostic biomarkers of this disease spectrum in blood, saliva, feces, cerebrospinal fluid (CSF), muscle tissue, and nerve tissue.

A five-year prospective and retrospective cohort study is evaluating blood and cerebrospinal fluid (CSF) samples from participants that include patients with ALS, AD, and PD as well as healthy subjects (NCT03217396). The levels of miRNAs, cytokines, chemokines, cell growth factors, neuronal damage markers, and mitochondrial and free d-amino acids from the collected biological specimens are being probed. Genotyping studies are being performed to identify single nucleotide polymorphisms (SNPs) in coding or regulatory regions of genes (miRNAs or proteins) involved in disrupting synaptic transmission of multiple sclerosis, a neurodegenerative disease that is comparable to dementia. Collectively, the goal is to identify potential biomarkers that can detect progressive synaptic damage associated with loss of sensory, motor, and cognitive functions in neurodegenerative conditions. In another observational study (NCT03388242), miRNA and protein expression in the blood were measured in order to differentiate between normal controls, patients with mild cognitive impairment, and patients with AD. miRNA and protein signatures were generated with the goal of developing sensitive and selective diagnostics.

Based on published reports and data, a few clinical studies are targeting specific miRNAs as potential diagnostics. An integral role for miR-206 in the pathomechanism of AD was previously reported, and therefore was a focus in a clinical trial investigating the olfactory neuroepithelial tissue in AD patients (NCT02129452). Olfactory neuroepithelial tissue is both easily accessible in a simple biopsy and is known to reflect brain pathology, which supports the confirmation of AD diagnosis. Studies have shown that beta-amyloid and tau proteins have been found in the olfactory neuroepithelium, which correlates with AD diagnosis. By analyzing the concentration of beta-amyloid and tau proteins using ELISA and miRNA-206 with RT-PCR, there is a possibility of finding pathological evidence of AD in olfactory neuroepithelial tissue for early diagnosis.

In addition to miR-206, miR-107 is associated with AD pathogenesis. A five-year prospective study from plasma of patients with mild cognitive impairment and those diagnosed with AD focused on miRNAs as a diagnostic marker, specifically miR-107 (NCT05055310). As an observational study, the miRNA concentration was measured over the duration of the clinical trial to determine its potential as a biomarker. As the putative clinical role for miRNAs in AD expands, analysis of high throughput data is intensifying. One group recently identified a panel of four stable miRNAs in the plasma of AD patients that could serve as endogenous controls to normalize results from large expression profile studies, potentially leading to enhanced accuracy [101].

The effect of miRNAs on AD is not limited to observational studies. An interventional study with Gemfibrozil examined a putative regulatory network of the drug with miR-107 (NCT02045056). Gemfibrozil is a lipid-lowering drug previously shown to reduce Aβ plaque formation and enhance memory in AD mouse models [102]. This clinical trial focused on 48 cognitively intact individuals and 24 individuals with early cognitive decline, with subjects randomly chosen to receive Gemfibrozil or the placebo. After 48 weeks, miR-107 levels were measured in both serum and CSF, and beta-amyloid 1–40 and 1–42 levels were measured in CSF. Previous reports discovered a signaling network between miR-107 and 𝛽-site amyloid precursor protein-cleaving enzyme (BACE1), leading to decreased miR-107 levels in AD patients. Additionally, miR-107 was of specific relevance as its levels were decreased in patients with early-stage AD [21,103].

## 6. Conclusions

The translation of miRNA candidates to the clinic is an active area of investigation in the biomedical and scientific communities. The pervasiveness of ncRNA transcripts in our genome underscores the critical role these molecules play in every human condition and disease, including top global health challenges such as cancer, heart disease, and dementia. The influence of miRNA networks has even been reported in relation to the recent COVID-19 pandemic with respect to the impact of the SARS-CoV-2 virus on neuroinflammatory responses that are linked to cognitive impairment. The pleiotropic regulation of critical gene networks by miRNA transcripts supports their promise as therapeutics, and their presence in patient biofluid samples indicates their potential as viable clinical biomarkers and diagnostic tools.

The expanding investigation of miRNAs in neuropathogenesis has uncovered unique miRNA transcripts and miRNA families That are implicated in dementia-related pathogenesis. These neurodegenerative processes are provoked through a range of pathways, including viral, inflammatory, toxic aggregates of Aβ and tau, and insulin-mediated mechanisms. A developing focus on miRNA networks linked to shared phenotypes across the spectrum of dementia-associated diseases, along with disease-specific miRNA patterns, is yielding new opportunities for clinical management. Although clinical trials investigating miRNAs and dementia to date have focused solely on the efficacy of miRNAs as biomarkers or diagnostics, their future as therapeutics remains open to exploration as their mechanisms become increasingly well defined.

## Figures and Tables

**Table 1:** Subsets of miRNAs associated with dementia-related disease.

AD	FTD	AD	VaD	DLB	PD	HD	CJD
let-7i-5p	miR-124	let-7i-5p	miR-17-5p	miR-3122	miR-9	miR-128a	let-7i-5p
miR-15a-5p	miR-134	miR-15a-5p	miR-18a-5p	miR-6861-3p	miR-34a	miR-10b-5p	miR-16-5p
miR-29c-3p	miR-99a	miR-29c-3p	miR-21-5p	miR-4298	miR-34b		miR-93-5p
miR-590-5p	miR-101	miR-590-5p	miR-29a-3p	miR-4728-5p	miR-34c		
miR-142-5p	miR-29b	miR-142-5p	let-7a-5p	miR-5698	miR-101		
miR-194-5p	miR-34a-5p	miR-194-5p	miR-10b	miR-1909-5p	miR-132		
miR-193b	miR-345-5p	miR-193b	miR-130b-3p	miR-451a			
miR-135a	miR-200c-3p	miR-135a		miR-21-5p			
miR-384	miR-10a-3p	miR-384		miR-23a-3p			
miR-132		miR-132		miR-126-3p			
miR-200b-5p		miR-200b-5p		let-7i-5p			
miR-1271		miR-1271		miR-151a-3p			
miR-142-5p		miR-142-5p		miR-133b			
let-7g-5p		let-7g-5p		miR-34a			
let-7d-5p		let-7d-5p		miR-137			
miR-342-5p		miR-342-5p		miR-7			

**Table 2:** Examples of plasma miRNAs dysregulated across dementia types.

miRNA	Reference for Dysregulation in AD	Reference for Dysregulation in VaD	Reference for Dysregulation in FTD
miR-502-3p	[54]	[55]	[56]
miR-451a	[54]	[55]	[56]
miR-127-3p	[57]	[55]	[57]

**Table 3:** Putative miRNA mechanisms and dementia.

Inflammatory	Viral	Gene Networks	Aβ and tau
miR-10b	miR-10b	miR-10a-5p	miR-298
miR-9	miR-1202	miR-100-5p	miR-23b-3p
miR-34a	CvmiR-5-5p	miR-22-3p	
miR-125b		miR-204-5p	
miR-146a		miR-26a	
miR-155		miR-203	
miR-22			
miR-17-5p			
miR-21-5p			
miR-126-3p			

**Table 4:** Brief descriptions of each clinical trial referenced in the section above.

Clinical Trial Name	Clinical Trial Number	Study Type	Phase	Disease/Disorder Investigated	Method
Explore Biomarkers of Motor Neuron Disease/Frontal Dementia Spectrum Disease in China	NCT04961450	Observational	Enrolling by Invitation	Frontotemporal Dementia Amyotrophic Lateral Sclerosis Motor Neuron Disease	Testing for biomarkers, including specific protein, miRNA, and DNA, in blood, saliva, feces, cerebrospinal fluid, muscle tissue, and nerve tissue
The Mechanism of MicroRNAs Network in Alzheimer’s Disease (MicroRNAs AD)	NCT05055310	Observational	Completed	Alzheimer’s Disease	Diagnostic Tests for miRNAs; Focus on miRNA-107
Biomarkers of Synaptic Damage in Multiple Sclerosis	NCT03217396	Observational	Recruiting	Multiples Sclerosis Parkinson Disease Amyotrophic Lateral Sclerosis Alzheimer’s Disease	Lumbar puncture; testing CSF concentrations for neurofilaments, beta amyloid, tau protein, inflammatory cytokines, and microRNAs
Protein and microRNA Markers for Early Detection of Alzheimer’s Disease	NCT03388242	Observational	Unknown	Alzheimer’s Disease	Testing blood samples for changes in miRNAs and proteins every six months for 1.5 years
Olfactory Neuroepithelial Tissue of Alzheimer Disease	NCT02129452	Observational	Completed	Alzheimer’s Disease	Testing the olfactory neuroepithelium for beta-amyloid protein, tau protein and micro-RNA 206 concentration
Modulation of Micro-RNA Pathways by Gemfibrozil in Predementia Alzheimer Disease	NCT02045056	Interventional	Early Phase One	Alzheimer’s Disease	Drug: Gemfibrozil Monitoring: miRNA-107 levels in Serum and CSF and Beta-amyloid levels in CSF
